# Development of a novel risk signature revealing prognostic and tumor microenvironmental features in breast cancer

**DOI:** 10.1097/MD.0000000000041369

**Published:** 2025-01-31

**Authors:** Yong Shen, Binbin Jiang, Yingbo Luo, Zhiwei Zhang

**Affiliations:** a Department of Breast Surgery, The Second Affiliated Hospital, Zhejiang University School of Medicine, Hangzhou, Zhejiang, China; b Cancer Institute (Key Laboratory of Cancer Prevention and Intervention, China National Ministry of Education, Key Laboratory of Molecular Biology in Medical Sciences, Zhejiang Province), The Second Affiliated Hospital, School of Medicine, Zhejiang University, Hangzhou, Zhejiang, China; c Department of Pathology, Hangzhou Fuyang Women and Children Hospital, Hangzhou, Zhejiang, China; d Department of Breast Surgery, Hangzhou Fuyang Women and Children Hospital, Hangzhou, Zhejiang, China.

**Keywords:** breast cancer, drug susceptibility, immune microenvironment, nomogram, pyrimidine metabolism

## Abstract

This study aimed to devise a breast cancer (BC) risk signature for based on pyrimidine metabolism-related genes (PMRGs) to evaluate its prognostic value and association with drug sensitivity. Transcriptomic and clinical data were retrieved from The Cancer Genome Atlas database and Gene Expression Omnibus repository. Pyrimidine metabolism-associated genes were identified from the Molecular Signatures Database collection. A risk signature was constructed through Cox regression and Lasso regression methods. Further, the relationship between the PMRG-derived risk feature and clinicopathological characteristics, gene expression patterns, somatic mutations, drug susceptibility, and tumor immune microenvironment was thoroughly investigated, culminating in the development of a nomogram. PMRGs displayed differential expression and diverse somatic mutations in BC. Univariate Cox analysis identified 36 genes significantly associated with BC prognosis, leading to the categorization of 2 BC molecular subtypes with discernible differences in prognosis. Using Lasso Cox regression, a risk signature composed of 16 PMRGs was established, wherein high-risk scores were indicative of poor prognosis. The PMRG-derived risk feature was also related to chemotherapy regimens and showed significant correlations with sensitivity to multiple drugs. Furthermore, distinct tumor immune microenvironment properties, gene expression profiles, and somatic mutation patterns were evident across varying risk scores. Ultimately, a nomogram was constructed incorporating the PMRGs-based risk signature alongside stage, and chemotherapy status, demonstrating excellent performance in prognosis prediction. We successfully developed a PMRG-based BC risk signature that effectively combines with clinicopathological attributes for accurate prognosis assessment in BC.

## 1. Introduction

Breast cancer (BC) is a leading cause of morbidity and mortality among women worldwide, and in the United States, it is estimated to account for 313,510 new cases and 42,780 deaths in 2024.^[1]^ As a heterogeneous disease characterized by diverse molecular profiles, it presents with varying degrees of invasiveness, progression potential, and responsiveness to therapy.^[2]^ Despite advances in early detection methods and personalized treatments,^[3]^ accurate prediction of patient outcomes remains challenging, necessitating the development of robust prognostic models that can guide therapeutic strategies.^[4]^ The identification of biomarkers associated with BC prognosis is thus an active area of research aimed at improving risk stratification, enabling tailored treatment regimens, and ultimately enhancing patient survival.^[5,6]^

Pyrimidine metabolism plays a pivotal role in cellular proliferation, particularly in rapidly dividing cells such as those found in tumors.^[7]^ This metabolic pathway supplies the building blocks for DNA and RNA synthesis, which are essential for cell growth and division.^[8]^ Aberrations in pyrimidine metabolism have been well-documented in various cancers, including BC,^[9]^ where they contribute to uncontrolled tumor growth. Dysregulation may manifest as increased de novo synthesis or altered catabolism, leading to accumulation of key metabolites and promoting oncogenic processes like angiogenesis, resistance to apoptosis, and genomic instability.^[10]^ Notably, several enzymes involved in this pathway have been implicated as potential therapeutic targets and prognostic biomarkers in multiple malignancies.^[11,12]^

**Figure 1.**
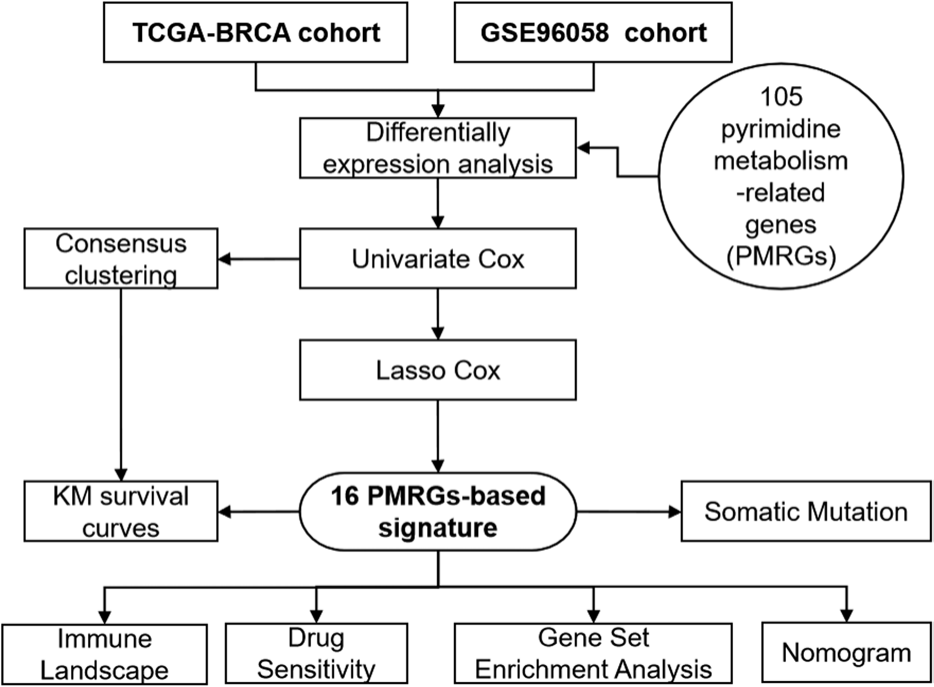
The workflow diagram of the study. BRCA=Breast Invasive Carcinoma, PMRG=pyrimidine metabolism-related gene, TCGA=The Cancer Genome Atlas.

This study aims to explore the potential utility of pyrimidine metabolism-related genes (PMRGs) in predicting BC prognosis. By leveraging large-scale transcriptomic data from public databases such as The Cancer Genome Atlas (TCGA), we systematically investigate the expression patterns and functional roles of PMRGs in BC tumorigenesis and progression. Ultimately, the goal is to construct and validate a novel prognostic model based on these genes that can better classify patients with BC into distinct risk groups. This predictive model has the potential to refine our understanding of BC biology and provide clinicians with a valuable tool to make more informed decisions regarding patient management and treatment planning. Figure 1 illustrated the workflow of this study.

## 2. Materials and methods

### 2.1. Data acquisition and processing

For the Breast Invasive Carcinoma (BRCA) study, we procured RNA sequencing data along with exhaustive clinical-pathological parameters from TCGA repository. Standardized RNA-seq expression quantification datasets and corresponding clinical metadata were accessed via TCGA’s Genomic Data Commons Data Portal at the URL (https://portal.gdc.cancer.gov/). Instances devoid of crucial clinical details, including overall survival data, prognosis status, initial diagnosis age, TNM stage, and lymph node involvement, were discarded. Moreover, in pursuit of robust survival analysis, cases exhibiting a follow-up period of fewer than 30 days were exempted from the analytical cohort to mitigate bias emanating from brief observation periods. For validation purposes and to broaden the sample spectrum, we also extracted the openly accessible GSE96058 dataset from the Gene Expression Omnibus (https://www.ncbi.nlm.nih.gov/geo/) archive, comprising transcriptomic profiles coupled with relevant clinical-pathological attributes of 3406 patients with BC. The GSE14999 dataset, containing gene expression profiles of 68 BC and adjacent normal tissues, was also downloaded from the Gene Expression Omnibus database for validation. A list of 105 PMRGs was curated from the Molecular Signatures Database (https://www.gsea-msigdb.org/gsea/msigdb/index.jsp) (Table S1, Supplemental Digital Content, http://links.lww.com/MD/O329).

### 2.2. Consensus clustering analysis

PMRGs with statistically significant differential expression (adjusted *P* value < 0.05 & |log2 (fold change)|>1) were identified utilizing the edgeR package.^[13]^ PMRGs demonstrating a significant association with BC prognosis in univariate Cox regression (*P* < .05) were selected for subsequent consensus clustering. The ConsensusClusterPlus package^[14]^ was applied to perform this clustering operation, employing the Partitioning Around Medoids algorithm and the “pearson” distance metric, succeeded by Kaplan–Meier survival analyses to discern prognostic disparities across derived clusters.

### 2.3. Development of a prognostic model

To construct a predictive risk signature, a Least Absolute Shrinkage and Selection Operator Cox regression model was fitted using the glmnet package. The risk score calculation was formulated as follows: risk score = Σ(βi × expi), where βi signifies the coefficient attributed to gene i and expi represents its respective expression level. Subsequently, based on the median risk score value, patients were stratified into high-risk and low-risk categories, followed by Kaplan–Meier survival analysis and receiver operating characteristic (ROC) curve assessment to gauge the predictive performance of the model.

### 2.4. Tumor microenvironment profiling

An extensive evaluation of the tumor microenvironment (TME) was carried out utilizing the IOBR package,^[15]^ which integrates a suite of algorithms designed to quantify immune cell infiltration within tumors. These methodologies encompass prominent computational techniques such as CIBERSORT, EPIC, xCell, MCP-counter, ESTIMATE, TIMER, quanTIseq, and immunophenoscore, thereby providing a multifaceted view of immune composition in the TME.

### 2.5. Pharmacological response prediction

Drug response analysis was conducted to infer the sensitivity of BC samples from the TCGA-BRCA cohort to a panel of 45 chemotherapeutic agents. Leveraging the pRRophetic package,^[16]^ drug sensitivity predictions were made through the invocation of the pRRopheticPredict() function, with the tissue specificity parameter set universally (“all”). In addition, batch effects inherent in the data were mitigated by applying the Combat algorithm to ensure enhanced accuracy and comparability of the sensitivity estimates across different experimental batches.

### 2.6. Transcriptomic differential expression and functional enrichment analyses

Differential gene expression scrutiny was executed utilizing the edgeR package, subsequently followed by gene set enrichment analysis (GSEA) facilitated by the clusterProfiler package.^[17]^ This GSEA process encompassed the interrogation of both gene ontology annotations and Kyoto Encyclopedia of Genes and Genomes pathways to decipher enriched biological processes and pathways linked to the observed expression changes.

### 2.7. Nomogram construction and performance assessment

A stepwise approach involving univariate and multivariate Cox proportional hazards regressions was taken to ascertain the prognostic significance of the calculated risk score alongside other clinicopathological features. Statistically significant independent predictors (*P* < .05) were then integrated into the construction of a nomogram using the rms package, aiming to estimate the 1-, 2-, and 3-year overall survival probabilities for patients with BC. The predictive accuracy and discriminatory power of the developed nomogram were thoroughly examined through ROC curve analysis, calibration curve assessment, and decision curve analysis–the latter implemented using the rmda package to determine clinical usefulness.

### 2.8. Validation of the PMRGs expression

The expression data of risk score-associated PMRGs for cell lines derived from breast tissue were obtained from the Cancer Cell Line Encyclopedia (CCLE) database (https://sites.broadinstitute.org/ccle/datasets, accessed on November 26, 2024). The expression differences of PMRGs associated with risk signatures between BC tissues and adjacent normal tissues in the GSE14999 cohort were compared using the Wilcoxon rank-sum test.

### 2.9. Statistical analyses

All statistical analyses and graphical representations were performed utilizing the R programming environment, version 4.3.2. For the examination of intergroup variations, the nonparametric Wilcoxon rank-sum test was applied, with a threshold of statistical significance set at *P* ≤ .05. In the context of survival analyses, Kaplan–Meier survival distributions were graphically depicted, and their differences were statistically tested through the log-rank test, implementing the functionalities offered by packages such as survival and survminer.

## 3. Results

### 3.1. PMRGs-derived BC molecular subtypes

**Figure 2.**
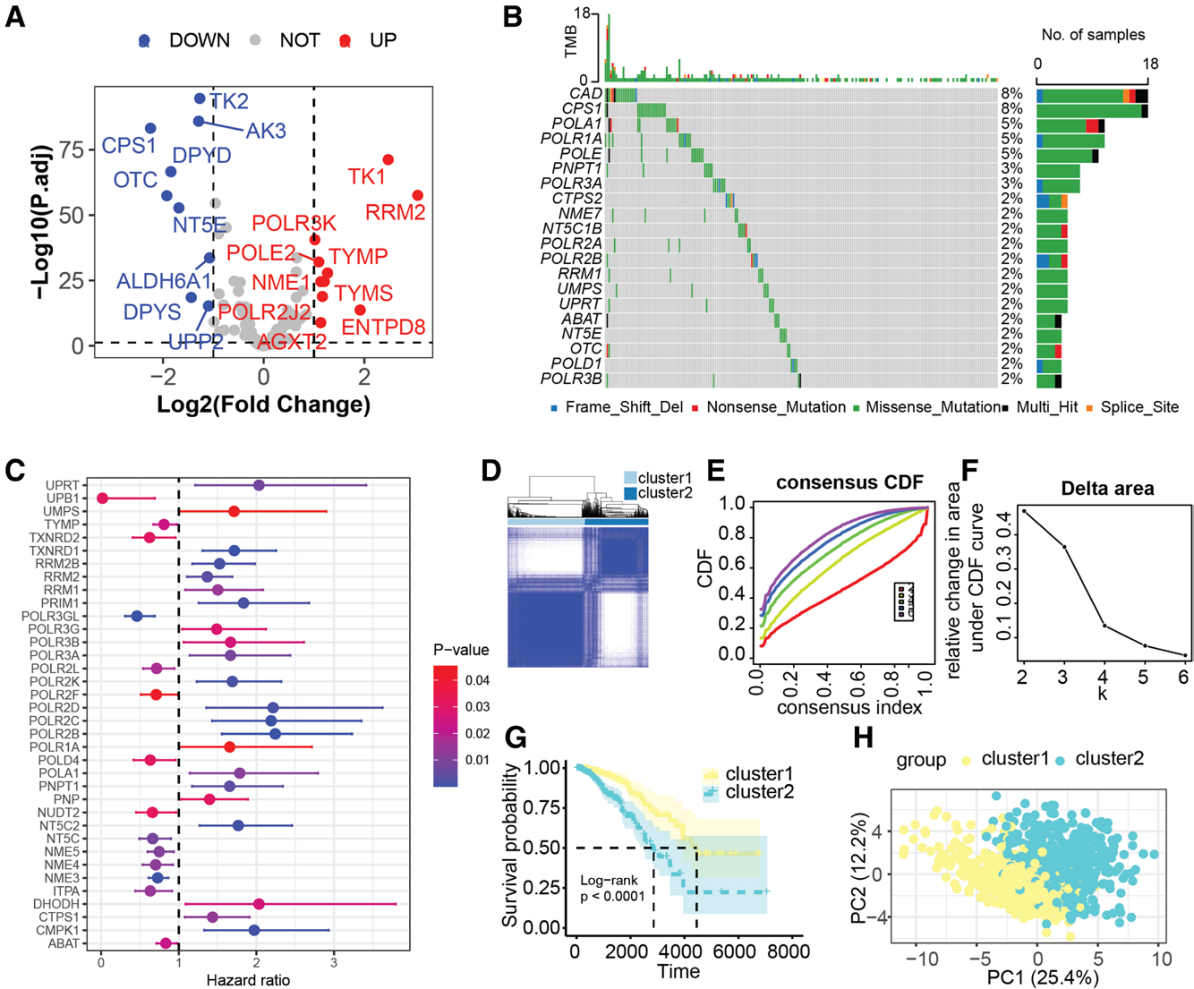
Expression, mutation, and prognostic implications of PMRGs in the TCGA-BRCA cohort. (A) Volcano plot depicting differential expression of PMRGs in breast cancer. (B) Oncoplot showing somatic mutations in PMRGs across breast cancer cases. (C) Hazard ratios for PMRGs significantly associated with prognosis. (D through F) Consensus clustering based on prognosis-related PMRGs classifies breast cancer into 2 distinct molecular subtypes, Cluster 1 and Cluster 2. (G) Kaplan–Meier survival analysis comparing the 2 clusters. (H) Principal component analysis of the prognosis-related PMRGs delineating the separation between Cluster 1 and Cluster 2. BRCA=Breast Invasive Carcinoma, PMRG=pyrimidine metabolism-related gene, TCGA=The Cancer Genome Atlas.

Among the pool of 105 PMRGs under investigation, 19 were identified as differentially expressed in BC, with 10 showing significantly upregulated expression and 9 demonstrating significantly downregulated expression (Fig. 2A). Figure 2B highlights the top 10 most frequently mutated genes within these PMRGs, with CAD and CPS1 presenting the highest frequencies of somatic mutations. The application of Univariate Cox regression analysis revealed that 36 of these PMRGs displayed significant associations with BC prognosis (*P* < .05, Fig. 2C), wherein 22 were classified as risky genes conferring detrimental outcomes, while 14 were protective genes (Table S2, Supplemental Digital Content, http://links.lww.com/MD/O329). Consensus clustering analysis, based on the expression patterns of these prognosis-related PMRGs, led to the segregation of patients with BC into 2 distinct molecular subtypes, designated Cluster 1 and Cluster 2 (Fig. 2D through 2F). Importantly, patients in Cluster 1 exhibited a more favorable prognosis compared to those in Cluster 2 (Fig. 2G). Principal component analysis further demonstrated a clear demarcation between Cluster 1 and Cluster 2 based on the expression profiles of these genes (Fig. 2H). These findings collectively suggest that PMRGs hold promise as potential biomarkers in BC, playing potentially pivotal roles in the pathogenesis and progression of the disease.

### 3.2. Constraction and evaluation of PMRG-derived risk signature

**Figure 3.**
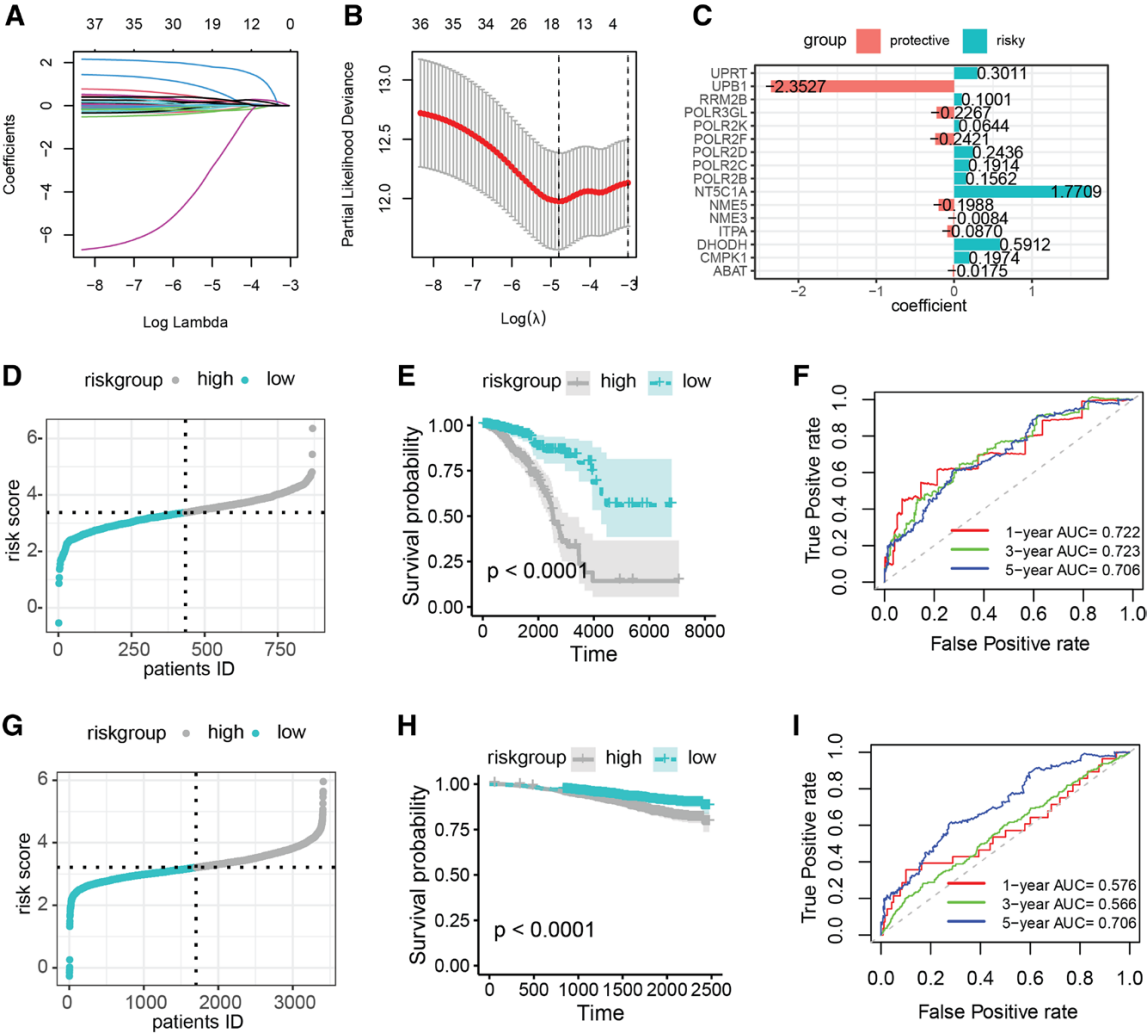
Development of a breast cancer prognostic risk model based on PMRGs. (A and B) Lasso Cox regression analysis identifies 16 PMRGs for constructing the risk signature. (C) Distribution of coefficients for the 16 PMRGs included in the risk model. (D through F) Stratification of the TCGA-BRCA cohort into high- and low-risk groups based on the PMRG risk signature, followed by Kaplan–Meier survival analysis and time-dependent ROC curve analysis. (G through I) Similar analyses for the GSE96058 cohort, partitioned by the same risk signature. BRCA=Breast Invasive Carcinoma, PMRG=pyrimidine metabolism-related gene, ROC=receiver operating characteristic, TCGA=The Cancer Genome Atlas.

Further, we employed Lasso Cox regression to scrutinize the prognosis-related PMRGs, resulting in the identification of a subset of 16 genes (Fig. 3A and 3B). Figure 3C elucidates the coefficients assigned to these genes within the risk signature, which were used to compute individual patient-specific risk scores. Upon stratifying the TCGA-BRCA cohort into high-risk and low-risk groups based on these scores (Fig. 3D), Kaplan–Meier survival analysis revealed a significantly improved prognosis for the low-risk group relative to the high-risk group (*P* < .0001, Fig. 3E). ROC analysis showed that the risk score achieved area under the curve (AUC) values of 0.722, 0.723, and 0.706 for predicting 1-, 2-, and 3-year overall survival rates in the TCGA-BRCA cohort, respectively (Fig. 3F). Consistently, in the independent GSE96058 cohort, patients categorized in the low-risk group also demonstrated superior outcomes when compared to those in the high-risk group (Fig. 3G and 3H). The discriminative power of the risk score for predicting 1-, 2-, and 3-year overall survival in this external dataset was evidenced by AUC values of 0.578, 0.588, and 0.708, respectively (Fig. 3I). Collectively, these results substantiate the effectiveness of the PMRG-derived risk signature in discriminating between patients with BC with unfavorable and favorable prognoses.

### 3.3. Clinical relevance of the PMRG-derived risk signature in BC patients

**Figure 4.**
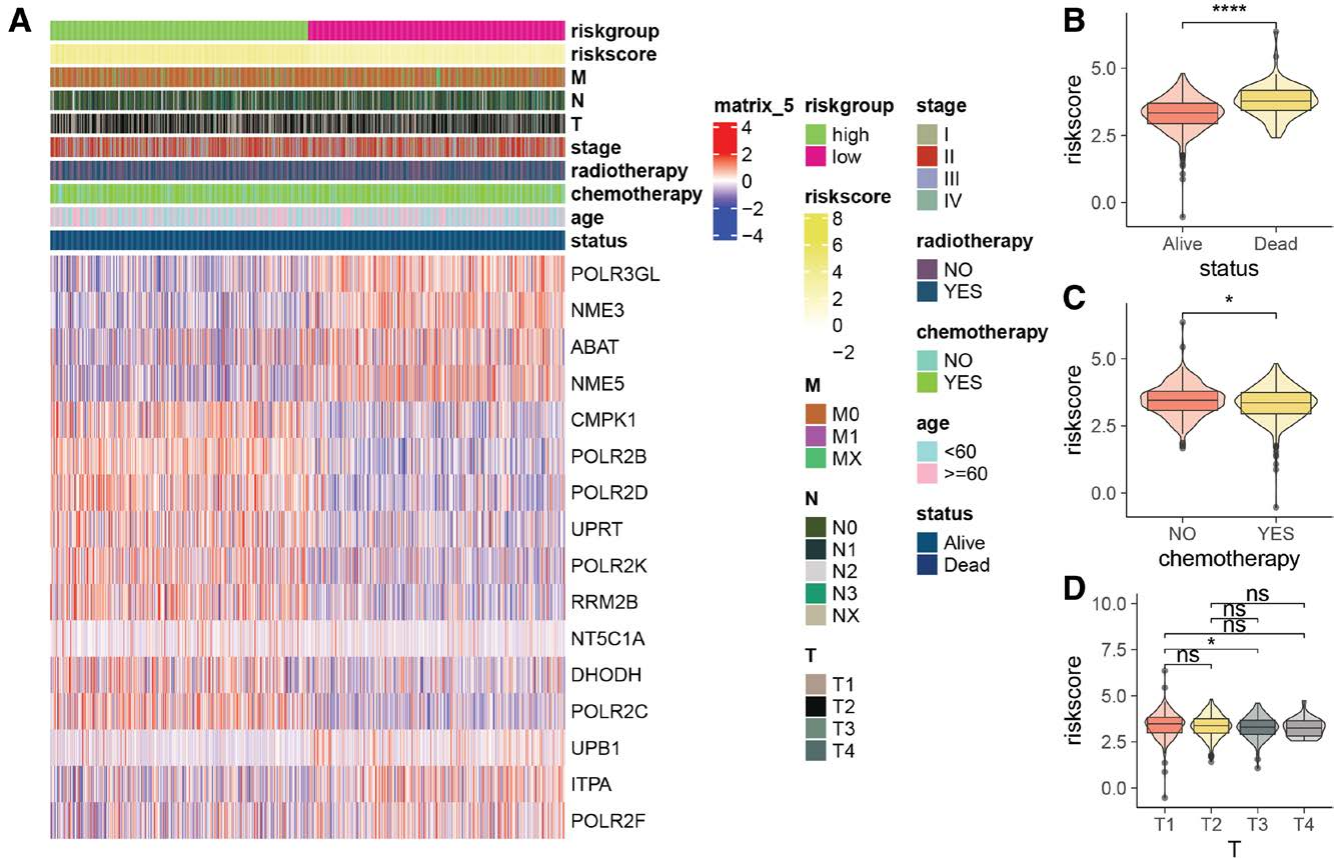
Association of the PMRG-derived risk score with clinicopathological characteristics. (A) Heatmap representing the expression of PMRGs along with clinical annotations. Comparison of risk score among patients with different (B) status categories, (C) chemotherapy histories, and (D) T-stages. PMRG=pyrimidine metabolism-related gene.

Figure 4A presents a heatmap depicting the expression patterns of the genes constituting the risk signature, with 7 genes overexpressed and 9 genes underexpressed. A striking contrast emerged upon comparing the risk scores of deceased patients, which were significantly elevated in comparison to those of alive patients (Fig. 4B). In addition, it was observed that untreated patients had significantly higher risk scores than their treated counterparts receiving chemotherapy (Fig. 4C). However, contrary to expectations, patients in the early T1 stage demonstrated higher risk scores than those in the advanced T3 stage (Fig. 4D).

### 3.4. PMRG-derived risk signature correlated with drug sensitivity profiles in BC

**Figure 5.**
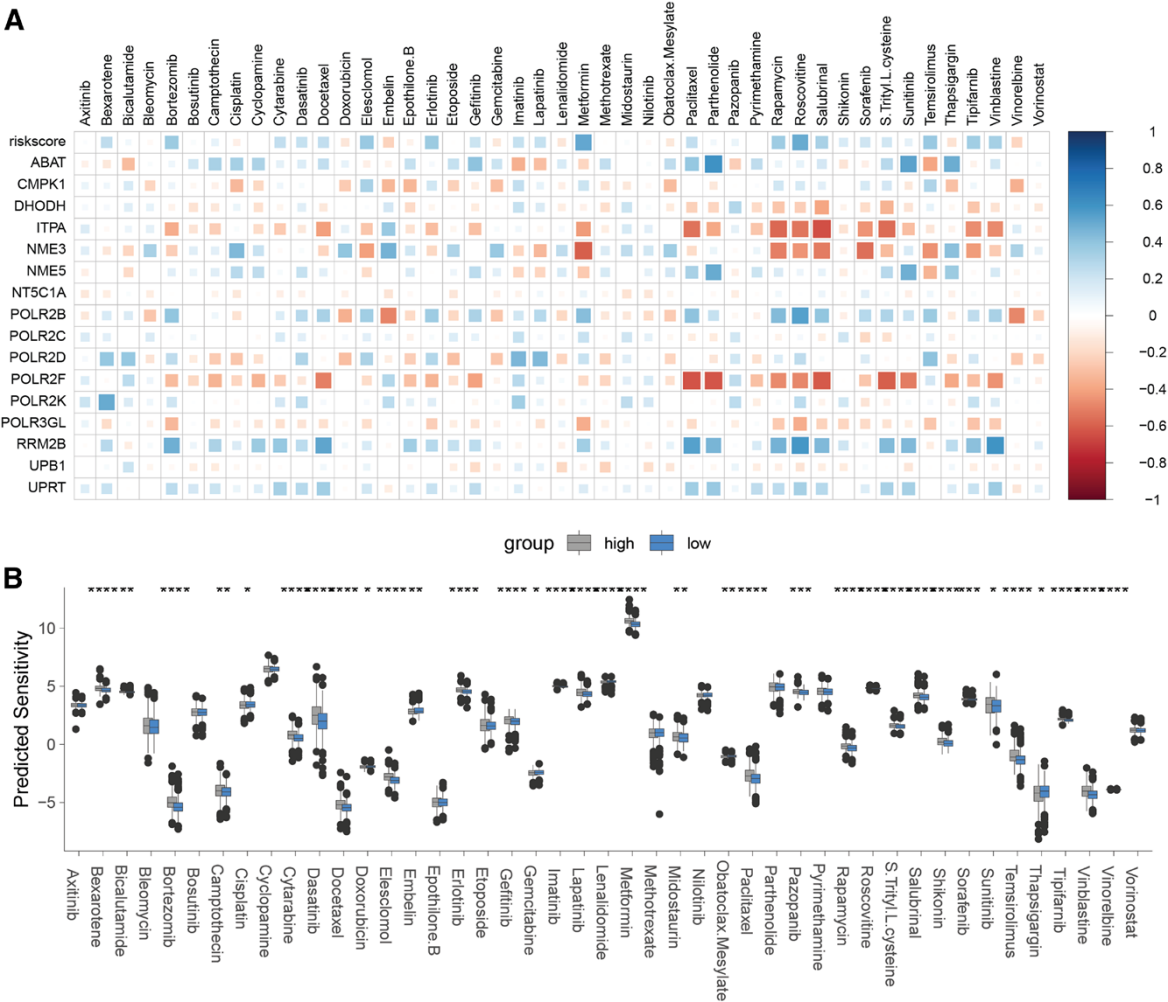
Relationship between PMRG-derived risk signature and drug sensitivity. (A) Heatmap illustrating the correlation between risk score, its component genes, and drug sensitivity. (B) Comparison of drug sensitivity between high- and low-risk groups. Asterisks denote statistical significance: *P < .05, **P < .01, ***P < .001, and ****P < .0001. PMRG=pyrimidine metabolism-related gene.

We undertook an extensive evaluation of the TCGA-BRCA cohort’s sensitivity to a panel of 45 chemotherapeutic agents and systematically investigated the relationship between the risk score and its constituent genes with respect to drug sensitivities. The analysis disclosed that the risk score, as well as individual genes ABAT, UPRT, RRM2B, POLR2K, and POLR2B, showed positive correlations with sensitivity to a majority of the drugs tested (Fig. 5A). Conversely, ITPA, POLR2F, and POLR3GL were negatively correlated with sensitivity to many of these agents. Upon comparing the drug sensitivity profiles between the low-risk and high-risk groups defined by the PMRG-based signature, we detected significant differences in sensitivity to 33 of the 45 drugs examined (Fig. 5B). These findings imply a potential role for PMRGs in mediating drug responsiveness, suggesting that the risk score could serve as a predictor of chemotherapy efficacy and possibly guide treatment strategies in patients with BC.

### 3.5. PMRG-derived risk signature correlated with tumor immune microenvironment in BC

**Figure 6.**
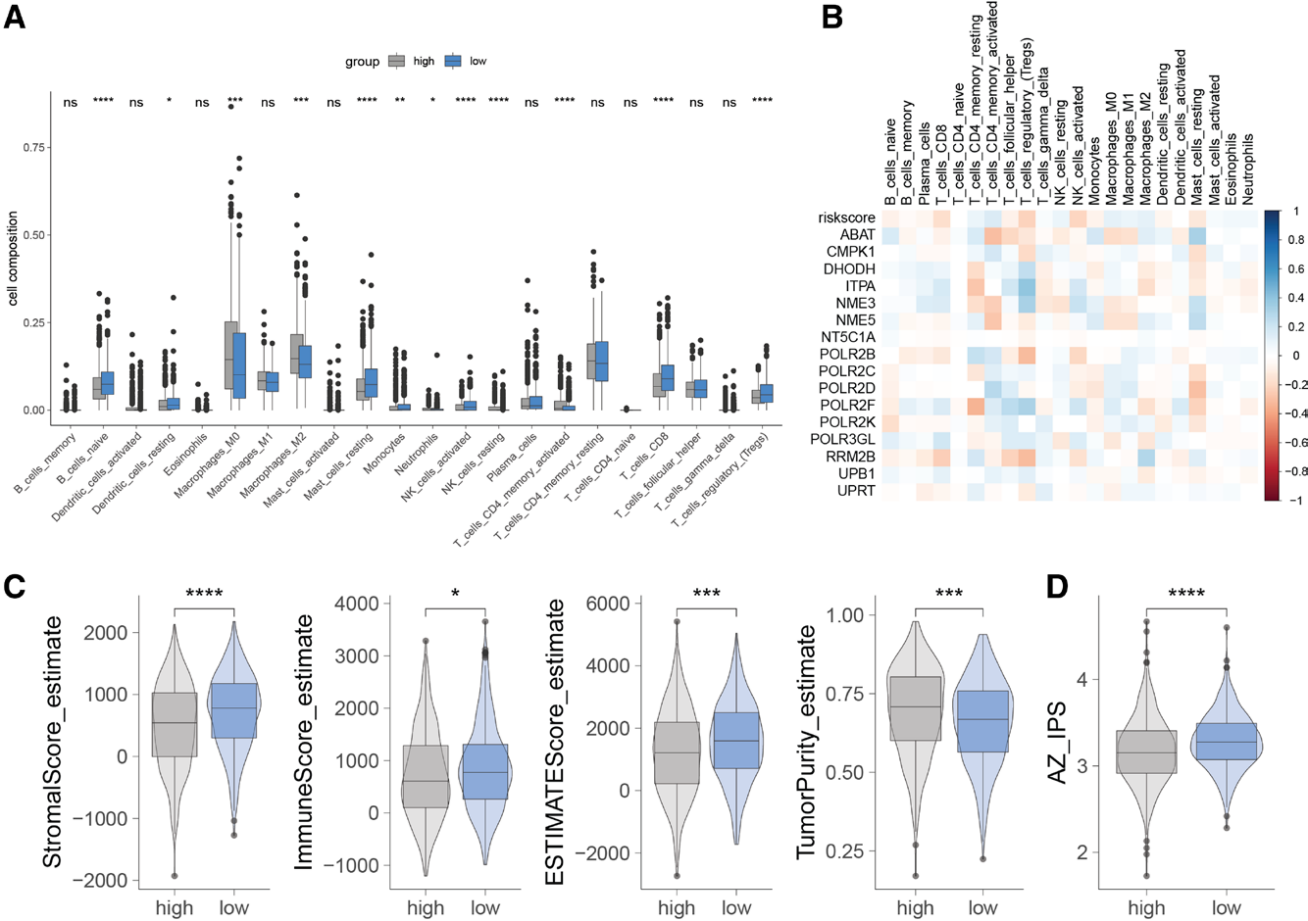
Link between the PMRG-derived risk signature and the tumor microenvironment. (A) Immune cell infiltration comparison between high- and low-risk groups. Asterisks indicate statistical significance: *P < .05, **P < .01, ***P < .001, and ****P < .0001. (B) Heatmap showing correlations between the risk score and immune cell infiltration. (C) Comparison of Stromalscore, Immunescore, EstimateScore, and Tumor Purity between high- and low-risk groups. (D) Differences in IPS scores between high- and low-risk groups. Asterisks denote statistical significance: *P < .05, ***P < .001, and ****P < .0001. IPS = immunophenoscore, PMRG=pyrimidine metabolism-related gene.

We conducted a thorough evaluation of the immune cell infiltration landscape across 22 immune cell types within the TCGA-BRCA cohort. Comparative analysis revealed that the high-risk group, as defined by the PMRG-based risk signature, exhibited a distinctive immunophenotype, characterized by significantly higher levels of M0 and M2 macrophages, resting natural killer (NK) cells, and activated memory CD4+ T cells, while concurrently displaying lower levels of naive B cells, resting dendritic cells, resting mast cells, monocytes, neutrophils, activated NK cells, CD8+ T cells, and regulatory T cells compared to the low-risk group (Fig. 6A). Correlation analyses illuminated a significant association between the risk score and the infiltration levels of T cells, NK cells, and macrophages (Fig. 6B), highlighting the interconnectedness of the PMRG-driven risk signature with immune cell populations within the tumor milieu. Moreover, when examining the tumor microenvironment components, we found that the high-risk group had lower stromal and immune scores, indicating reduced stromal and immune cell content, and conversely, a higher tumor purity compared to the low-risk group (Fig. 6C). In addition, the high-risk group demonstrated lower immunophenoscore than the low-risk group, as shown in Figure 6D. Taken together, these findings provide compelling evidence that the PMRG-derived risk signature is intricately linked to the immune microenvironment in BC, influencing the balance of immune infiltrates and potentially shaping the tumor’s response to immunotherapy and other targeted treatments.

### 3.6. Distinct gene expression and mutation patterns between high- and low-risk groups

**Figure 7.**
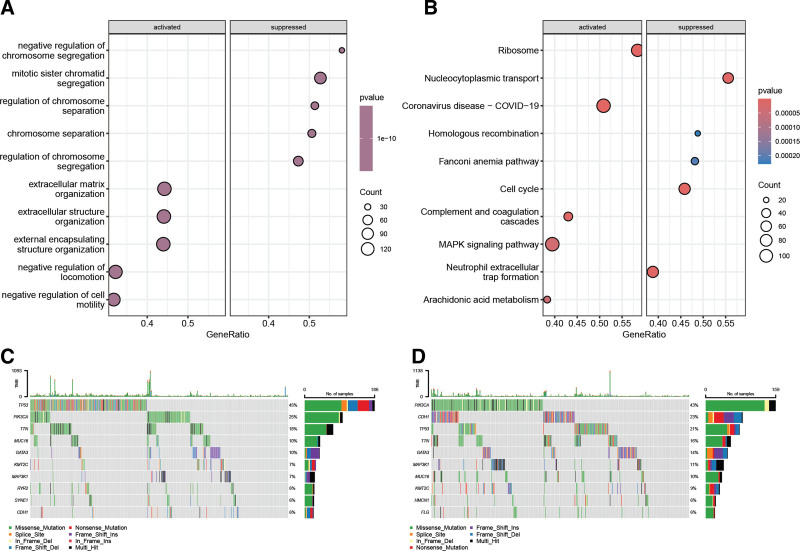
Distinctive expression and somatic mutation profiles between high- and low-risk groups. (A) Significantly suppressed and activated gene ontologies in the high-risk group compared to the low-risk group. (B) KEGG pathways significantly activated or inhibited in the high-risk group versus the low-risk group. (C) Top 10 most frequently mutated genes in the high-risk group. (D) Top 10 most frequently mutated genes in the low-risk group. KEGG=Kyoto Encyclopedia of Genes and Genomes.

GSEA uncovered distinctive functional signatures between high- and low-risk groups as defined by the PMRG-derived risk classification. In the high-risk group, there was significant activation of biological processes pertinent to extracellular matrix organization, negative regulation of locomotion, and cell motility (Fig. 7A). Conversely, pathways integral to chromosome segregation were notably suppressed in this group. Moreover, pathways such as ribosome biogenesis, complement and coagulation cascades, mitogen-activated protein kinase signaling, and arachidonic acid metabolism were found to be significantly upregulated in the high-risk group (Fig. 7B). On the other hand, pathways essential for nucleocytoplasmic transport, cell cycle progression, and neutrophil extracellular trap formation were downregulated. Comparative analysis of mutational profiles revealed discrepancies between the high- and low-risk cohorts. Notably, the gene CDH1 displayed a wider prevalence of mutations in the low-risk group (Fig. 7C and 7D). These results collectively demonstrate distinct transcriptional and mutational landscapes between the high- and low-risk groups, providing insights into the underlying mechanisms contributing to their differing clinical phenotypes and potentially guiding the development of targeted therapies.

### 3.7. Nomogram integrating PMRG-derived risk signature and clinicopathological features

**Figure 8.**
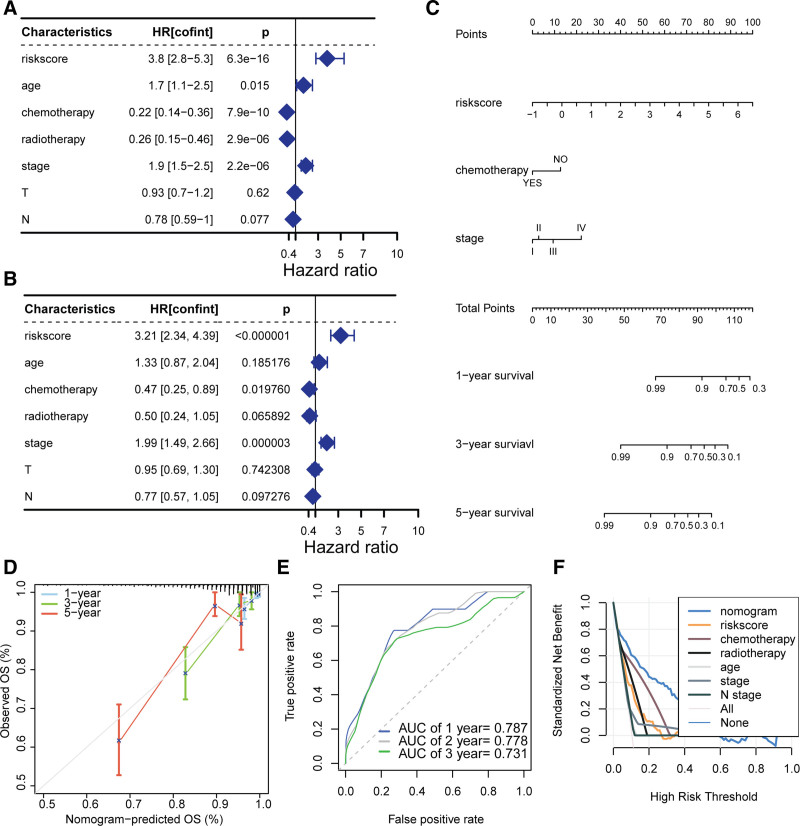
Construction and validation of a nomogram. (A) Univariate and (B) Multivariate Cox regression analyses of the PMRG-derived risk score and clinicopathological parameters. (C) Nomogram incorporating risk score, chemotherapy, and stage for predicting 1-, 2-, and 3-year overall survival. (D) Calibration curve analysis demonstrating agreement between nomogram-predicted and observed survival. (E) Time-dependent ROC curve analysis assessing predictive accuracy. (F) Decision curve analysis comparing the standardized net benefit of the nomogram against other prognostic factors. PMRG=pyrimidine metabolism-related gene, ROC=receiver operating characteristic.

Following extensive univariate and multivariate Cox regression analyses, we determined that the risk score, age, chemotherapy, radiotherapy, and tumor stage were all significantly associated with BC prognosis (Fig. 8A and 8B). However, after adjusting for confounding variables, only the risk score, chemotherapy, and tumor stage emerged as independent prognostic indicators for BC. Based on these findings, we proceeded to construct a nomogram integrating these 4 independent predictors to facilitate the estimation of 1-, 2-, and 3-year overall survival probabilities for patients with BC (Fig. 8C). The calibration plot indicated excellent concordance between the nomogram-predicted and actual (observed) survival times, as evidenced by the close approximation of predicted probabilities to the ideal 45-degree line (Fig. 8D), thus affirming the nomogram’s accurate calibration. ROC curve analysis demonstrated that the nomogram achieved high discriminatory ability, with AUC values of 0.787, 0.778, and 0.731 for the prediction of 1-, 2-, and 3-year overall survival, respectively (Fig. 8E). Decision curve analysis further supported the clinical utility of the nomogram, revealing a higher standardized net benefit compared to other prognostic factors specifically in predicting 1-year overall survival (Fig. 8F). This underscores the practical advantage of using the nomogram as a decision support tool in clinical practice for patients with BC.

### 3.8. Validation of PMRG expression in BC

**Figure 9.**
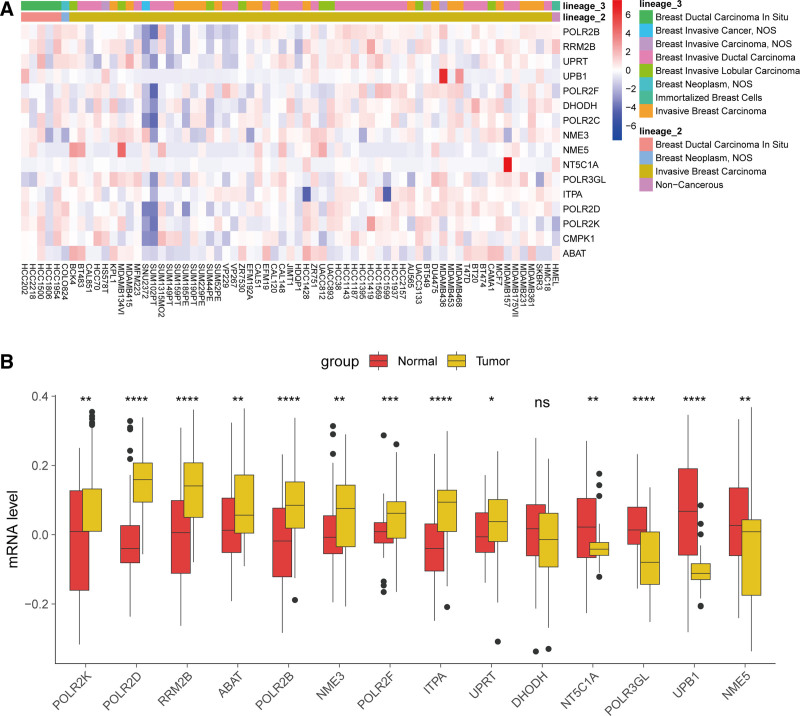
Validation of PMRG expression. (A) Heatmap showing the expression of 16 PMRGs associated with risk signatures in breast cancer cell lines from the CCLE database. (B) Comparison of PMRG expression associated with risk signatures between breast cancer tissues and adjacent normal tissues in the GSE14999 cohort. *P < .05, **P < .01, ***P < .001, and ****P < .0001. CCLE=Cancer Cell Line Encyclopedia, PMRG=pyrimidine metabolism-related gene.

To further validate the expression of PMRGs associated with risk signatures in BC, we utilized the CCLE and GSE14999 cohorts for analysis. Figure 9A shows the expression of 16 PMRGs across various breast tissue cell lines in the CCLE. Among these, UPRT, UPB1, POLR2F, POLR2C, NME5, POLR3GL, POLR2D, POLR2K, and CMPK1 exhibited lower expression in noncancerous cell lines, while the remaining genes showed higher expression. However, these genes displayed higher heterogeneity in their expression in cancer cell lines. In the GSE14999 cohort, we obtained the expression profiles of 14 PMRGs associated with risk signatures. Upon comparison, we found that POLR2D, POLR2K, RRM2B, ABAT, POLR2B, NME3, POLR2F, ITPA, and UPRT were significantly upregulated in cancer tissues, whereas NT5C1A, POLR3GL, UPB1, and NME5 were significantly downregulated (Fig. 9B). The expression of DHODH did not show significant changes.

## 4. Discussion

In prior research, PMRGs have been successfully employed to develop cancer prognostic risk models^[18]^; however, herein, we innovatively constructed a PMRG-based BC risk signature rooted in the expression profiles of 16 PMRGs, which effectively stratifies patients with BC according to their prognosis. Notably, this novel signature exhibits a significant correlation with drug sensitivity, an aspect not extensively explored in previous studies,^[19]^ thus shedding light on the potential role of PMRGs in chemoresistance. Moreover, our risk signature outperforms comparable models reported earlier in terms of predictive power.^[19]^

The PMRG-derived risk signature reflects the widespread involvement of these genes in tumorigenesis, progression, and chemoresistance mechanisms. For instance, CMPK1 encodes a protein pivotal for deoxynucleotide biosynthesis, essential for DNA replication and repair. Overexpression or mutation of CMPK1 can disrupt DNA synthesis, promoting tumor initiation and progression in BC and other malignancies.^[20,21]^ DHODH plays a critical role in the pyrimidine metabolic pathway, and its abnormal expression or activity alterations might influence tumor cell proliferation. Studies have shown that DHODH is implicated in tumor growth, invasion, and resistance in various cancers, including BC.^[22,23]^ In specific cancer contexts, elevated expression or mutation of NT5C1A may aid tumor cells in evading DNA damage induced by chemotherapy, pointing to its potential role in BC chemoresistance development.^[24]^ RRM2B is indispensable for DNA replication and repair, contributing to DNA stability maintenance. Aberrations in RRM2B in breast and other cancers could diminish DNA damage repair efficiency, thereby affecting tumor radiosensitivity and chemotherapy responsiveness.^[25,26]^ Both UPB1 and UPRT participate in pyrimidine metabolism, where low UPB1 expression has been associated with poor prognosis in lung adenocarcinoma,^[27]^ and UPRT is involved in the activation of 5-fluorouracil, a commonly used chemotherapeutic agent.^[28]^ Changes in the expression and function of these genes in BC could potentially impact the response to chemotherapy drugs.^[29]^ Thus, the unique characteristics of the PMRG-derived risk signature in relation to prognosis and drug sensitivity likely stem from the biological functions of these genes.

Furthermore, PMRGs also exhibit associations with the tumor immune microenvironment (TIME), particularly involving T cells, NK cells, macrophages, and mast cells. Variations in the infiltration of these immune cells give rise to distinct TIME configurations, which ultimately influence the efficacy of immunotherapies and drug sensitivities.^[30–32]^ ABAT-mediated mitochondrial rewiring and receptor-dependent signaling responses are critical for T cell-mediated inflammation, playing roles in Th17 and regulatory T cell differentiation.^[33]^ Research suggests that inhibiting DHODH can impede clonal expansion of activated T cells and the expression of effector molecules; DHODH and mitochondria-linked pyrimidine synthesis serve as independent and significant cellular brakes for activated T cells.^[34]^ NME3 and NME5 – members of the NME family – are involved in diverse biological processes, including energy metabolism and signal transduction, which modulate signaling pathways relevant to the immune system and energy metabolism, thereby shaping the TME.^[35]^ Studies have shown that NME3 promotes inflammation downstream of TLR5 signaling, enhancing cancer immunotherapy.^[36]^ However, current findings do not fully elucidate the underlying mechanisms behind the correlations between PMRGs and the infiltration of various immune cells, necessitating more comprehensive experimental investigations in the future.

Ultimately, we successfully integrated the PMRG-based risk signature with other key clinical-pathological parameters to create a nomogram model intended to provide a more precise and comprehensive tool for patient with BC prognosis assessment. Despite this accomplishment, several limitations remain in our study. First, although we established the risk signature model using existing public databases, its validity requires prospective cohort validation to confirm its stability and accuracy in real-world clinical settings. Second, the detailed mechanisms by which PMRGs contribute to tumor resistance pathways and their influence on the TIME have yet to be fully elucidated. A series of biological experiments, such as in vitro and in vivo functional assays, immunohistochemistry, and single-cell sequencing, are needed to investigate how PMRGs specifically regulate tumor cell resistance and modulate the infiltration and function of various immune cells within the tumor.

## 5. Conclusion

In summary, we have developed and validated a novel PMRG-derived BC risk signature and elucidated its relationships with the tumor microenvironment and drug sensitivity, offering additional insights into tumor resistance mechanisms. The nomogram constructed based on the PMRG-derived risk signature, when combined with clinical-pathological features, presents a promising application for BC prognosis assessment, but broader prospective cohort validations are still required to substantiate its utility.

## Author contributions

**Conceptualization:** Yong Shen, Binbin Jiang, Yingbo Luo, Zhiwei Zhang.

**Data curation:** Yong Shen, Binbin Jiang, Yingbo Luo, Zhiwei Zhang.

**Formal analysis:** Yong Shen, Binbin Jiang, Yingbo Luo, Zhiwei Zhang.

**Funding acquisition:** Yong Shen, Binbin Jiang, Yingbo Luo, Zhiwei Zhang.

**Investigation:** Yong Shen, Binbin Jiang, Yingbo Luo, Zhiwei Zhang.

**Methodology:** Yong Shen, Binbin Jiang, Yingbo Luo, Zhiwei Zhang.

**Project administration:** Yong Shen, Binbin Jiang, Yingbo Luo, Zhiwei Zhang.

**Resources:** Yong Shen, Binbin Jiang, Yingbo Luo, Zhiwei Zhang.

**Software:** Yong Shen, Binbin Jiang, Yingbo Luo, Zhiwei Zhang.

**Supervision:** Yong Shen, Binbin Jiang, Yingbo Luo, Zhiwei Zhang.

**Validation:** Yong Shen, Binbin Jiang, Yingbo Luo, Zhiwei Zhang.

**Visualization:** Yong Shen, Binbin Jiang, Yingbo Luo, Zhiwei Zhang.

**Writing – original draft:** Yong Shen, Binbin Jiang, Yingbo Luo, Zhiwei Zhang.

**Writing – review & editing:** Yong Shen, Binbin Jiang, Yingbo Luo, Zhiwei Zhang.

## Supplementary Material


